# Correction: Lapatinib-Binding Protein Kinases in the African Trypanosome: Identification of Cellular Targets for Kinase-Directed Chemical Scaffolds

**DOI:** 10.1371/journal.pone.0092488

**Published:** 2014-03-26

**Authors:** The PLOS ONE Staff

## Notice of Republication

This article was republished on February 21, 2014 to replace an incorrectly published version. Please download this article again to view the correct version. The originally published, uncorrected article and the republished, corrected article are provided here for reference.

## Supporting Information

File S1 Originally published, uncorrected article.
(PDF)

File S2 Republished corrected article.
(PDF)
