# Supplementary material for: Lapatinib-Binding Protein Kinases in the African Trypanosome: Identification of Cellular Targets for Kinase-Directed Chemical Scaffolds
Source: PLoS One. 2013 Feb 20;8(2):e56150. doi: 10.1371/journal.pone.0056150 (PMC3577790; doi:10.1371/journal.pone.0056150)
Supplement: Table S1 — Properties of Peptides Used to Identify Protein Protein Kinases. (DOCX) [file pone.0056150.s001.docx]

**Supplementary Table 1.** Properties of Peptides Used to Identify Protein kinases Eluted with Drugs

**Lapatinib**

| ***Protein*** | ***Peptides*** | ***Peptide Sequence*** | ***Residue no.*** | ***Obs m/z*** | ***Pep. ID score*** |
| --- | --- | --- | --- | --- | --- |
| Tb927.4.5180 (TbLBPK1) | 6 | K.QQQQDLNHEK.K | 280-291 | 626.2482 | 0.9968 |
|  |  | R.RDEVEELK.K | 220-229 | 509.6981 | 0.6855 |
|  |  | K.AFDLQEAR.Y | 336-345 | 475.6574 | 0.9954 |
|  |  | R.RPFAEGESQQQIWQNK.L | 537-554 | 649.9718 | 0.9907 |
|  |  | R.QLTM'QLEELSVR.R | 208-221 | 732.4445 | 0.8381 |
|  |  | K.Q^ATLPSYGLVNDTAVFR.K | 92-110 | 918.8546 | 0.9974 |
|  |  |  |  |  |  |
| Tb927.5.800 (TbLBPK2) | 4 | K.TRHPQLAFEAR.F | 45-57 | 442.9836 | 0.9613 |
|  |  | R.GTNIQTGDPVAIK.L | 27-41 | 657.6895 | 0.9991 |
|  |  | K.TTLM'LAEQM'IAR.I | 108-121 | 706.2448 | 0.7625 |
|  |  | R.GSLPWQGLK.A | 213-223 | 493.7395 | 0.8633 |
|  |  |  |  |  |  |
| Tb927.3.1570 (TbLBPK3) | 2 | R.RPLSICDSPSLEAK.F | 118-133 | 525.1835 | 0.8942 |
|  |  | K.ASLFTDILPTAATLPK.R | 497-514 | 829.9172 | 0.9427 |
|  |  |  |  |  |  |
| Tb10.61.3140 (TbLBPK4) | 2 | R.VAGQGTFGTVQLAR.D | 24-39 | 703.4095 | 0.9918 |
|  |  | K.Q^PLPAEVYDLCGK.I | 276-290 | 736.8621 | 0.7407 |
|  |  |  |  |  |  |

**Canertinib**

| \| ***Protein*** \| ***Peptides*** \| ***Peptide Sequence*** \| ***Residue no.*** \| ***Obs m/z*** \| ***Pep. ID score*** \| \| --- \| --- \| --- \| --- \| --- \| --- \| \| Tb927.4.5180 (TbLBPK1) \| 8 \| K.QQQQDLNHEK.K \| 280-291 \| 626.2181 \| 0.9978 \| \|  \|  \| R.RDEVEELKK.T \| 220-230 \| 573.7622 \| 0.8786 \| \|  \|  \| R.EVWVEGNK.M \| 198-207 \| 481.1006 \| 0.6751 \| \|  \|  \| K.AFDLQEAR.Y \| 336-345 \| 475.6469 \| 0.9924 \| \|  \|  \| R.RPFAEGESQQQIWQNK.L \| 537-554 \| 649.6866 \| 0.9782 \| \|  \|  \| R.VNDEDASAFVAVPALGHNGR.Y \| 299-320 \| 680.9972 \| 0.9951 \| \|  \|  \| K.QATLPSYGLVNDTAVFR.K \| 92-110 \| 926.9899 \| 0.9983 \| \|  \|  \| R.LIIM'QVVSALR.Y \| 426-438 \| 630.3447 \| 0.9996 \| \|  \|  \|  \|  \|  \|  \| \| Tb927.5.800 (TbLBPK2) \| 7 \| R.THQHIPYK.E \| 165-174 \| 512.6885 \| 0.8583 \| \|  \|  \| K.RIHDTLQEGR.A \| 298-309 \| 409.0963 \| 0.8124 \| \|  \|  \| K.TRHPQLAFEAR.F \| 45-57 \| 442.9318 \| 0.9897 \| \|  \|  \| R.GTNIQTGDPVAIK.L \| 27-41 \| 657.7538 \| 0.9991 \| \|  \|  \| R.YCSINTHIGIEQSR.R \| 183-198 \| 560.2397 \| 0.7294 \| \|  \|  \| K.TTLM'LAEQM'IAR.I \| 110-121 \| 705.7018 \| 0.9941 \| \|  \|  \| R.GSLPWQGLK.A \| 213-223 \| 493.4398 \| 0.8532 \| \|  \|  \|  \|  \|  \|  \| \| Tb927.3.1570 (TbLBPK3) \| 3 \| R.LAEQGLK.K \| 136-144 \| 380.0811 \| 0.8823 \| \|  \|  \| R.GDNTSGDWGYYK.R \| 198-211 \| 682.0219 \| 0.9837 \| \|  \|  \| K.ASLFTDILPTAATLPK.R \| 497-514 \| 830.2547 \| 0.9995 \| \|  \|  \|  \|  \|  \|  \| \| Tb10.61.3140 (TbLBPK4) \| 2 \| K.NYFYTVGGEGR.R \| 80-92 \| 631.8053 \| 0.9443 \| \|  \|  \| R.VAGQGTFGTVQLAR.D \| 24-39 \| 703.1091 \| 0.9980 \| \|  \|  \|  \|  \|  \|  \| \| Tb10.61.1880 (TbCBPK1) \| 2 \| K.LADFDQAK.V \| 154-163 \| 454.3133 \| 0.9479 \| \|  \|  \| K.GDNLLISM'DTGIAK.L \| 140-155 \| 732.5655 \| 0.9793 \| |  |  |  |  |  |
| --- | --- | --- | --- | --- | --- | --- | --- | --- | --- | --- | --- | --- | --- | --- | --- | --- | --- | --- | --- | --- | --- | --- | --- | --- | --- | --- | --- | --- | --- | --- | --- | --- | --- | --- | --- | --- | --- | --- | --- | --- | --- | --- | --- | --- | --- | --- | --- | --- | --- | --- | --- | --- | --- | --- | --- | --- | --- | --- | --- | --- | --- | --- | --- | --- | --- | --- | --- | --- | --- | --- | --- | --- | --- | --- | --- | --- | --- | --- | --- | --- | --- | --- | --- | --- | --- | --- | --- | --- | --- | --- | --- | --- | --- | --- | --- | --- | --- | --- | --- | --- | --- | --- | --- | --- | --- | --- | --- | --- | --- | --- | --- | --- | --- | --- | --- | --- | --- | --- | --- | --- | --- | --- | --- | --- | --- | --- | --- | --- | --- | --- | --- | --- | --- | --- | --- | --- | --- | --- | --- | --- | --- | --- | --- | --- | --- | --- | --- | --- | --- | --- | --- | --- | --- | --- | --- | --- | --- | --- | --- | --- | --- | --- | --- | --- | --- | --- | --- |

**AEE788**

| ***Protein*** | ***Peptides*** | ***Peptide Sequence*** | ***Residue no.*** | ***Obs m/z*** | ***Pep. ID score*** |
| --- | --- | --- | --- | --- | --- |
| Tb927.4.5180 (TbLBPK1) | 8 | K.Q^QQQDLNHEK.K | 280-291 | 626.3433 | 0.9859 |
|  |  | R.DAQIDELR.E | 115-124 | 480.3636 | 0.9918 |
|  |  | K.AFDLQEAR.Y | 336-345 | 475.2571 | 0.9952 |
|  |  | R.RPFAEGESQQQIWQNK.L | 537-554 | 649.8096 | 0.9828 |
|  |  | R.QLTM'QLEELSVR.R | 208-221 | 732.3815 | 0.9538 |
|  |  | R.VNDEDASAFVAVPALGHNGR.Y | 299-320 | 680.9680 | 0.9838 |
|  |  | K.QATLPSYGLVNDTAVFR.K | 92-110 | 927.2361 | 0.9975 |
|  |  | R.LIIM'QVVSALR.Y | 426-438 | 630.2559 | 0.9991 |
|  |  |  |  |  |  |
| Tb927.5.800 (TbLBPK2) | 4 | R.IEFVHSK.S | 120-128 | 430.6907 | 0.8105 |
|  |  | K.TRHPQLAFEAR.F | 45-57 | 442.8966 | 0.9938 |
|  |  | R.GTNIQTGDPVAIK.L | 27-41 | 657.5636 | 0.9991 |
|  |  | R.GSLPWQGLK.A | 213-223 | 493.4788 | 0.8568 |
|  |  |  |  |  |  |
| Tb927.3.1570 (TbLBPK3) | 5 | R.RGGGPETSPPR.G | 187-199 | 556.2838 | 0.9307 |
|  |  | R.RPLSICDSPSLEAK.F | 118-133 | 525.2563 | 0.9985 |
|  |  | R.LSNGEVVLEVENR.S | 439-453 | 730.2529 | 0.9727 |
|  |  | R.DLKPQNLLLTGR.S | 348-361 | 684.9254 | 0.8121 |
|  |  | K.ASLFTDILPTAATLPK.R | 497-514 | 830.4440 | 0.9994 |

Modifications:
Fixed
C 57.021
Variable
C^ -17.027
E^ -18.011
M' 15.995
Q^ -17.02
