# Supplementary material for: Correction: Lapatinib-Binding Protein Kinases in the African Trypanosome: Identification of Cellular Targets for Kinase-Directed Chemical Scaffolds
Source: PLoS One. 2014 Mar 26;9(3):e92488. doi: 10.1371/journal.pone.0092488 (PMC3966806; doi:10.1371/journal.pone.0092488)
Supplement: File S1 — Originally published, uncorrected article. (PDF) [file pone.0092488.s001.pdf]

OPEN ACCESS Freely available online

PLOS ONE

Lapatinib-Binding Protein Kinases in the African Trypanosome: Identification of Cellular Targets for Kinase-Directed Chemical Scaffolds

Samiksha Katiyar[1], Irina Kufareva[2], Ranjan Behera[1], Sarah M. Thomas[1], Yuko Ogata[4], Michael Pollastri[3], Ruben Abagyan[2]*, Kojo Mensa-Wilmot[1]*

1 Department of Cellular Biology, University of Georgia, Athens, Georgia, United States of America, **2** UCSD Skaggs School of Pharmacy and Pharmaceutical Sciences, La Jolla, California, United States of America, **3** Department of Chemistry and Chemical Biology, Northeastern University, Boston, Massachusetts, United States of America, **4** Proteomics Facility, Fred Hutchinson Cancer Research Center, Seattle, Washington, United States of America

Abstract

Human African trypanosomiasis is caused by the eukaryotic microbe *Trypanosoma brucei*. To discover new drugs against the disease, one may use drugs in the clinic for other indications whose chemical scaffolds can be optimized via a medicinal chemistry campaign to achieve greater potency against the trypanosome. Towards this goal, we tested inhibitors of human EGFR and/or VEGFR as possible anti-trypanosome compounds. The 4-anilinoquinazolines canertinib and lapatinib, and the pyrrolopyrimidine AEE788 killed bloodstream *T. brucei in vitro* with GI_{50} in the low micromolar range. Curiously, the genome of *T. brucei* does not encode EGFR or VEGFR, indicating that the drugs recognize alternate proteins. To discover these novel targets, a trypanosome lysate was adsorbed to an ATP-sepharose matrix and washed with a high salt solution followed by nicotinamide adenine dinucleotide (NAD^+). Proteins that remained bound to the column were eluted with drugs, and identified by mass spectrometry/bioinformatics. Lapatinib bound to Tb927.4.5180 (termed *T. brucei* lapatinib-binding protein kinase-1 (TbLBPK1)) while AEE788 bound Tb927.5.800 (TbLBPK2). When the NAD^+ wash was omitted from the protocol, AEE788, canertinib and lapatinib eluted TbLBPK1, TbLBPK2, and Tb927.3.1570 (TbLBPK3). In addition, both canertinib and lapatinib eluted Tb10.60.3140 (TbLBPK4), whereas only canertinib desorbed Tb10.61.1880 (TbCBPK1). Lapatinib binds to a unique conformation of protein kinases. To gain insight into the structural basis for lapatinib interaction with TbLBPKs, we constructed three-dimensional models of lapatinib•TbLBPK complexes, which confirmed that TbLBPKs can adopt lapatinib-compatible conformations. Further, lapatinib, AEE788, and canertinib were docked to TbLBPKs with favorable scores. Our studies (a) present novel targets of kinase-directed drugs in the trypanosome, and (b) offer the 4-anilinoquinazoline and pyrrolopyrimidines as scaffolds worthy of medicinal chemistry and structural biology campaigns to develop them into anti-trypanosome drugs.

Citation: Katiyar S, Kufareva I, Behera R, Thomas SM, Ogata Y, et al. (2013) Lapatinib-Binding Protein Kinases in the African Trypanosome: Identification of Cellular Targets for Kinase-Directed Chemical Scaffolds. PLoS ONE 8(2): e56150. doi:10.1371/journal.pone.0056150

Editor: Ziyin Li, University of Texas-Houston Medical School, United States of America

Received August 20, 2012; **Accepted** January 5, 2013; **Published** February 20, 2013

Copyright: © 2013 Katiyar et al. This is an open-access article distributed under the terms of the Creative Commons Attribution License, which permits unrestricted use, distribution, and reproduction in any medium, provided the original author and source are credited.

Funding: This work was supported by National Institutes of Health AI076647. The funders had no role in study design, data collection and analysis, decision to publish, or preparation of the manuscript.

Competing Interests: The authors have declared that no competing interests exist.

* E-mail: mensawil@uga.edu (KM-W); rabagyan@ucsd.edu (RA)

Introduction

Human African trypanosomiasis (HAT) is caused by the protozoan *Trypanosoma brucei*. New drugs for the treatment of HAT are needed as the current ones have significant issues relating to administration route, toxicity and/or drug resistance [1,2]. To assist drug discovery efforts, we must find novel small molecules that kill the parasite, and, in the long-term, also understand the mechanisms of their action in the trypanosome.

Protein phosphorylation on Tyr residues, catalyzed by protein Tyr kinases (PTKs) or dual-specificity protein kinases that act on Tyr as well as Ser/Thr [3–6], is important for cell signaling networks that control numerous processes [7]. PTKs in vertebrates exist in two general forms; receptor tyrosine kinases (RTKs) and non-receptor Tyr kinases (NRTKs). RTKs such as epidermal growth factor receptor (EGFR) or vascular endothelial growth factor receptor (VEGFR), are transmembrane proteins and are activated upon binding of hormones to their extracellular domains. NRTKs, *e.g.*, Src-family protein kinases, are cytosolic, and can be activated by RTKs or cytokine receptors.

Mechanistic studies of phosphotyrosine (pTyr) signaling in human cells led to discovery of drugs (*e.g.*, lapatinib, and imatinib) that are incredibly well-tolerated [8–10]. Lapatinib (GW572016) (GlaxoSmithKline) inhibits human EGFR/HER2 [11]. AEE788 (Novartis) inhibits EGFR and VEGFR [12,13], and canertinib (CI-1033) (Pfizer) is a pan-inhibitor of EGFRs [14–16].

T. brucei has a divergent pTyr system, as compared to its human host. Whereas Tyr-phosphorylated proteins are present [17,18], trypanosomes lack RTKs [19,20]. Consequently, Tyr-phosphorylation of proteins is projected to be performed by dual-specificity enzymes that act on Ser/Thr as well as Tyr residues [19]. Further, the trypanosome genome does not encode classic pTyr-binding domains (*e.g.*, SH2) [21]. The biological importance of Tyr-phosphorylation in *T. brucei* is inferred from the effect of small molecule Tyr kinase inhibitors; (i) genistein blocks trypanosome

replication [22]; (ii) Tyrphostin A47 inhibits interferon-γ induced Tyr phosphorylation of proteins [23], and (iii) Tyrphostin A47 inhibits receptor mediated endocytosis by the parasite [24,25].

Due to the uniqueness of the Tyr-phosphorylation system in the trypanosome it seems likely that a detailed understanding of pTyr signaling (*i.e.*, enzymes, substrates and pathways regulated) could yield great insight into novel signal transduction pathways in a deeply-diverged eukaryote, and lead to discovery of novel targets for lead drug discovery.

In the work reported here, we show that the 4-anilinoquinazolines lapatinib and canertinib kill bloodstream *T. brucei*. Since the trypanosome genome does not encode orthologs of EGFR/HER2, we searched for and identified novel trypanosome targets of the drugs, using an affinity chromatography protocol that involved protein elution with drugs: Proteins were identified with mass spectrometry and bioinformatics. This protocol which is easily adaptable for the discovering targets of kinase inhibitors in other cells, led to identification five novel binding-proteins of lapatinib, AEE788, and canertinib in *T. brucei*. The four putative targets of lapatinib were evaluated theoretically for their ability to bind the drug using homology modeling and molecular docking. Results from these studies support conclusions drawn from our ligand-affinity chromatography data.

Materials and Methods

Reagents

Immobilized γ-aminophenyl-ATP-sepharose was purchased from Jena Biosciences. NAD^+, and ATP were purchased from Sigma. Silver stain kit was obtained from Pierce. Lapatinib, canertinib, and AEE788 were gifts from GlaxoSmithKline, Pfizer, and Novartis, respectively.

Effects of Drugs on Trypanosome and HeLa Cell Replication

Bloodstream form *T. brucei brucei* Lister 427 were seeded at a density of 2×10^3 cells/ml and cultured in HMI-9 medium [26] in a 24 well plate. Two microliters of DMSO (control) or different concentrations of drugs (as specified in the Figure legends) from 200X DMSO stocks were added to the cultures. Cells were incubated at 37°C for 48 h, and counted with a haemocytometer.

HeLa cells were cultured in high glucose Dulbecco's modified Eagle's medium (Cellgro) supplemented with 10% FBS (Hyclone), 100 U/ml penicillin (Cellgro), 100 μg/ml streptomycin (Cellgro) [27]. Cells were cultured at 37°C with humidified 5% CO_2. For drug inhibition studies, cells were seeded in a 24 well plates at a density of 1×10^5 cells/ml. Drugs or DMSO were added in 2 μl up to 25 μM final concentration. Cells were incubated at 37°C for 48 h and counted with a haemocytometer.

Drug Affinity Chromatography of Proteins

T. brucei were cultured axenically [26] to a density of 1×10^6 cells/ml and harvested (2×10^8 total cells). After washing with 10 ml of cold BBS/G (buffered saline plus glucose; 50 mM bicine, 150 mM NaCl, 5 mM KCl, 1% glucose, pH 7.4) containing 1 mM sodium vanadate, cells were lyzed on ice in 1 ml of cold Buffer A (20 mM Tris-HCl pH 7.4, 60 mM $MgCl_2$, 60 mM KCl, 1 mM DTT, 0.2% NP-40, 1 mM PMSF, 2 μg/mL aprotinin, 5 μg/ml leupeptin, 37 μgγ/ml TLCK, 2 μM FMKO24, and 1 mM sodium vanadate). The cell lysate was incubated on ice for 20 minutes and centrifuged at 15,000×g for 10 min at 4°C. The supernatant was incubated for 18 h at 4°C with 50 μl (settled bed volume) of ATP-sepharose (Sepharose 4B was used as control in a separate experiment.) The resin was washed with 500 μl each of the following (i) buffer A; (ii) buffer A containing 1 M KCl, and finally (iii) buffer A. When specified, the resin was eluted twice with 100 μM NAD^+ (100 μM in buffer A).

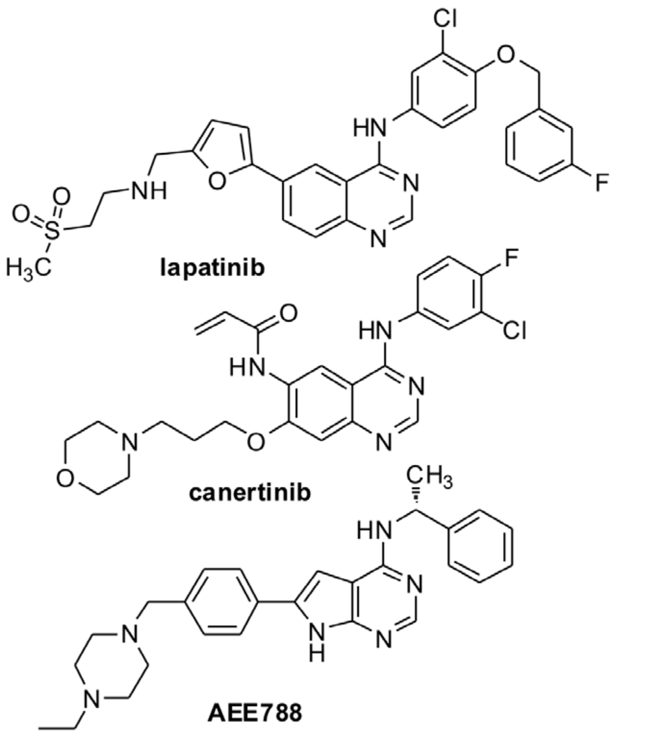

Figure 1. Chemical structure of lapatinib, AEE788, and canertinib.
doi:10.1371/journal.pone.0056150.g001

Several concentrations of drugs (i.e., 1 μM, 10 μM, and 100 μM) were used in trial experiments to elute proteins from the ATP-affinity column. For (subsequent) mass spectrometry analysis it is important that the bands detected by silver staining of protein be sufficiently dark. After analysis of the proteins eluted in several independent trial studies, we determined that 100 μM concentration was optimal for elution of proteins bound by lapatinib, AEE788 and canertinib: The drug concentration needed varies for each scaffold. For each drug, proteins bound to the affinity column were eluted twice (from different matrices) with 100 γl of lapatinib (100 γM) or AEE788 (100 μM) or canertinib (100 μM) (Drugs are diluted to their final concentrations in buffer A from DMSO stocks at 10 mM concentration). A control elution was performed with buffer A containing 1% DMSO, because the final concentration of that solvent in the drug mixtures was 1%, and eluted proteins identified by mass spectrometry. Eluted polypeptides were precipitated with trichloroacetic acid and pelleted (15,000×g for 10 min). The supernatant was aspirated, the precipitate was suspended in 2× SDS-PAGE sample buffer (10 μl), and the pH adjusted by adding 5–7 μl of 30% NH_4OH. Proteins were separated by SDS-PAGE (12%), and polypeptide bands detected by staining with silver. Each lane of gel was cut into four pieces each of which was minced with a sterile blade in preparation for trypsinization (see below).

In-gel Trypsin Digestion and Mass Spectrometry

Polyacrylamide gel pieces were destained with a kit (Invitrogen), and dehydrated with acetonitrile. Proteins were digested overnight with trypsin (5 ng/μl, Promega) in ammonium bicarbonate (50 mM) at 37°C. Peptides were extracted with formic acid (5%

v/v in water) for 30 min, and then with acetonitrile. Extracts were pooled, dried in a SpeedVac, and peptides were purified with ZipTip™ C18 chromatography (Millipore Corporation).

LC-MS/MS analysis was performed with a LTQ mass spectrometer (Thermo Scientific). The LC system was configured in a vented format consisting of a fused-silica nanospray needle packed in-house with Magic C18 AQ 100 Å reverse-phase media (Michrom Bioresources Inc.), and a trap containing Magic C18 AQ 200 Å [30]. Peptide samples were loaded onto the column and separated using a two-mobile-phase system consisting of 0.1% formic acid in water (A) and 0.1% acetic acid in acetonitrile (B). The mass spectrometer was operated in a data-dependent MS/MS mode over the m/z range of 400–1800. For each cycle, the five most abundant ions from the scan were selected for MS/MS analysis using 35% normalized collision energy. Selected ions were dynamically excluded for 45 seconds. Raw MS/MS data were submitted to the Computational Proteomics Analysis System (CPAS), a web-based system built on the LabKey Server v11.2 that utilizes Trans-Proteomic Pipeline v4.3 [28,29], and searched using X! Tandem engine (2009.10.01.1) against *T.brucei* protein database v. 4.0 (ftp://ftp.sanger.ac.uk/pub/databases/T.brucei_sequences/T.brucei_genome_v4/), which included additional common contaminants such as human keratin. The following modifications were considered: loss of water or ammonia from terminal glutamic acid or glutamine respectively, and oxidation of methionine. The mass tolerances were set ±2 Da and ±0.5 Da for precursor and fragment ions respectively. The enzyme was set to Trypsin, and up to 2 missed cleavages were permitted. The search output files were analyzed and validated by ProteinProphet [30,31]. Peptides matched with an error rate <0.05 were accepted, and corresponding proteins identified with two or more peptides are reported. Experiments were repeated thrice; proteins are reported only if they were detected in at least two separate experiments (Table 1). Properties of peptides used to identify protein kinases are presented in Table S1.

Structure-directed Sequence Alignment of Lapatinib-binding Pockets

All four trypanosome kinases have medium to high homology templates in PDB (Table 2). The closest homology templates for TbLBPK1 and TbLBPK3 in the PDB are a putative calcium-dependent protein kinase 1 from *Cryptosporidium parvum* (CpCDPK1, Uniprot ID A3FQ16, 34% sequence identity to TbLBPK1 in the kinase domain) and a putative calcium-dependent protein kinase 3 from *Toxoplasma gondii* (TgCDPK3, Uniprot ID Q3HNM6, 30% sequence identity to TbLBPK3 in the kinase domain). Despite belonging to CaMK family of Ser/Thr kinases, both of these templates are crystallized in lapatinib-compatible backbone conformations (PDB codes 2wei and 3hzt, respectively). In the case of TbLBPK2 and TbLBPK4, the closest homology templates human casein kinase Iδ (Uniprot ID KC1D_HUMAN) and a putative glycogen synthase kinase from *Leishmania major* (Uniprot ID Q4QE15), respectively, are crystallized in an active-like, and therefore lapatinib-incompatible conformation. Thus, the closest template models of TbLBPK1 and TbLBPK3 were capable of favorably docking lapatinib while the closest template for TbLBPK2 and TbLBPK4 could not. We hasten to add that the lapatinib-conformation is dynamic; it is an alternate to the enzyme active-conformation. Therefore, its absence from an x-ray structure does not preclude its existence; in some instances presence of the drug might stabilize lapatinib-compatible conformations in select protein kinases.

Given this background (above), we enhanced modeling based on closest homology templates with more distant modeling protocols using

Table 1. Protein kinases eluted with drugs after 1 M KCl wash of the affinity column.

Lapatinib

Protein	*Peptides*	*Peptide Sequence*	*Residue no.*
Tb927.4.5180 (TbLBPK1)	6	K.QQQQDLNHEK.K	280–291
		R.RDEVEELK.K	220–229
		K.AFDLQEAR.Y	336–345
		R.RPFAEGESQQQIWQNK.L	537–554
		R.QLTM'QLEELSVR.R	208–221
		K.Q^ATLPSYGLVNDTAVFR.K	92–110
Tb927.5.800 (TbLBPK2)	4	K.TRHPQLAFEAR.F	45–57
		R.GTNIQTGDPVAIK.L	27–41
		K.TTLM'LAEQM'IAR.I	108–121
		R.GSLPWQGLK.A	213–223
Tb927.3.1570 (TbLBPK3)	2	R.RPLSICDSPSLEAK.F	118–133
		K.ASLFTDILPTAATLPK.R	497–514
Tb10.61.3140 (TbLBPK4)	2	R.VAGQGTFGTVQLAR.D	24–39
		K.Q^PLPAEVYDLCGK.I	276–290

Canertinib

Protein	*Peptides*	*Peptide Sequence*	*Residue No.*
Tb927.4.5180 (TbLBPK1)	8	K.QQQQDLNHEK.K	280–291
		R.RDEVEELKK.T	220–230
		R.EVWVEGNK.M	198–207
		K.AFDLQEAR.Y	336–345
		R.RPFAEGESQQQIWQNK.L	537–554
		R.VNDEDASAFVAVPALGHNGR.Y	299–320
		K.QATLPSYGLVNDTAVFR.K	92–110
		R.LIIM'QVVSALR.Y	426–438
Tb927.5.800 (TbLBPK2)	7	R.THQHIPYK.E	165–174
		K.RIHDTLQEGR.A	298–309
		K.TRHPQLAFEAR.F	45–57
		R.GTNIQTGDPVAIK.L	27–41
		R.YCSINTHIGIEQSR.R	183–198
		K.TTLM'LAEQM'IAR.I	110–121
		R.GSLPWQGLK.A	213–223
Tb927.3.1570 (TbLBPK3)	3	R.LAEQGLK.K	136–144
		R.GDNTSGDWGYYK.R	198–211
		K.ASLFTDILPTAATLPK.R	497–514
Tb10.61.3140 (TbLBPK4)	2	K.NYFYTVGGEGR.R	80–92
		R.VAGQGTFGTVQLAR.D	24–39
Tb10.61.1880 (TbCBPK1)	2	K.LADFDQAK.V	154–163
		K.GDNLLISM'DTGIAK.L	140–155

AEE788

Table 1. Cont.

**Lapatinib**			
Protein	***Peptides***	***Peptide Sequence***	***Residue no.***
Protein	***Peptides***	***Peptide sequence***	***Residue No.***
Tb927.4.5180 (TbLBPK1)	8	K.Q^QQQDLNHEK.K	280–291
		R.DAQIDELR.E	115–124
		K.AFDLQEAR.Y	336–345
		R.RPFAEGESQQQIWQNK.L	537–554
		R.QLTM'QLEELSVR.R	208–221
		R.VNDEDASAFVAVPALGHNGR.Y	299–320
		K.QATLPSYGLVNDTAVFR.K	92–110
		R.LIIM'QVVSALR.Y	426–438
Tb927.5.800 (TbLBPK2)	4	R.IEFVHSK.S	120–128
		K.TRHPQLAFEAR.F	45–57
		R.GTNIQTGDPVAIK.L	27–41
		R.GSLPWQGLK.A	213–223
Tb927.3.1570 (TbLBPK3)	5	R.RGGGPETSPPR.G	187–199
		R.RPLSICDSPSLEAK.F	118–133
		R.LSNGEVVLEVENR.S	439–453
		R.DLKPQNLLLTGR.S	348–361
		K.ASLFTDILPTAATLPK.R	497–514

Proteins presented were detected at least twice in three independent affinity chromatography/mass spectrometry analyses.

doi:10.1371/journal.pone.0056150.t001

structures of human EGFR in complex with lapatinib (PDB 1xkkA [32]) as templates, since our affinity chromatography studies identified TbLBPKs as lapatinib-binding polypeptides. These structures provided a variety of suitable backbone conformations of the kinase domains for the modeling studies. Sequence identity between EGFR and TbLBPKs is between 18% and 22%, which is below the 40% cutoff for reliable homology modeling [33–35]. Consequently, sequence alignment methods failed to find the correct residue correspondence in several regions of the kinase domain. We resolved the alignment ambiguities by constructing structural alignments of EGFR to the closest homology templates of TbLBPKs in PDB (Table 1) followed by propagation of the obtained residue correspondence onto the target sequences via a sequence-based alignment. From these, lapatinib-binding was discerned.

Homology Modeling of TbLBPK Structures

The structures of TbLBPK1-4 and TbCBPK1 were built using the homology modeling platform of the Internal Coordinate Mechanics (ICM) software [36–38]. Initially, well-aligned regions of the target kinase were threaded through the backbone coordinates of the template structure. Loops, insertions and deletions were searched against a large database of PDB fragments for similar sequence and termini topology; well-scoring fragments were incorporated into the nascent model and minimized in its context. Template hydrogen bonds were converted into distance restraints; the model side-chains were thoroughly sampled to find the global minimum of the energy function that included soft van der Waals, electrostatic, hydrogen bonding, torsional strain, and distance restraint terms. To resolve the remaining steric conflicts, the model was subjected to gradient minimization with both side-chain and backbone variables relaxed. The final models were evaluated using two methods: first, ICM Protein Health evaluation confirmed the absence of torsional or steric inaccuracies; second, PROCHECK [39] evaluation placed at least 80% of the residues in the most favorable regions on the Ramachandran plot with at least 97% being in the allowed regions. Visual inspection of the remaining (unfavorable) residues confirmed that they located in extended loops distant from the lapatinib binding site and were therefore not influential in the docking studies.

Ligand Docking and Scoring

ICM ligand docking is based on biased probability Monte Carlo optimization of the ligand internal coordinates in the set of grid potential maps that represent the protein binding pocket. A diverse set of ligand conformers was first generated from 2D coordinates by 2D to 3D conversion and thorough sampling *in vacuo*. The conformers were placed into the binding pocket in four principal orientations and used as starting points for Monte Carlo optimization. The optimized energy function included the ligand internal strain and a weighted sum of the grid map values in ligand atom centers. The top-scoring ligand poses were merged with the kinase models to obtain full-atom models of the complexes which were further evaluated with full-atom ICM ligand binding score [40]. The score was calculated by:

$$S_{bind} = E_{int} + T\Delta S_{Tor} + E_{vw} + a_1 E_{el} + a_2 E_{hb} + a_3 E_{hp} + a_4 E_{sf}$$

where E_{vw}, E_{el}, E_{hb}, E_{hp}, and E_{sf} are Van der Waals, electrostatic, hydrogen bonding, non-polar and polar atom solvation energy

Table 2. TbLBPKs1-4 and their structural homologs in PDB.

**Protein Kinase**	**System ID**	**UniProt ID**	**Kinase Domain**	**Closest PDB**	**homologs**
				**SwissProt ID**	**Sequence Identity to Target (%)**
**TbLBPK1**	Tb927.4.5180	Q584I8	320:601	SNF1_YEAST A3FQ16_CRYPV	31 34
**TbLBPK2**	Tb927.5.800	Q57W25	11:282	KC1D_HUMAN	69
**TbLBPK3**	Tb927.3.1570	Q57XZ0	213:488	MARK2_HUMAN B6KR85_TOXGO	32 30
**TbLBPK4**	Tb10.61.3140	Q388M1 (GSK3γ)	20:310	Q4QE15_LEIMA GSK3B_HUMAN	69 54
**TbCBPK1**	Tb10.61.1880	Q388C6	16:284	M3K5_HUMAN PAK1_HUMAN	37.5 37.3

doi:10.1371/journal.pone.0056150.t002

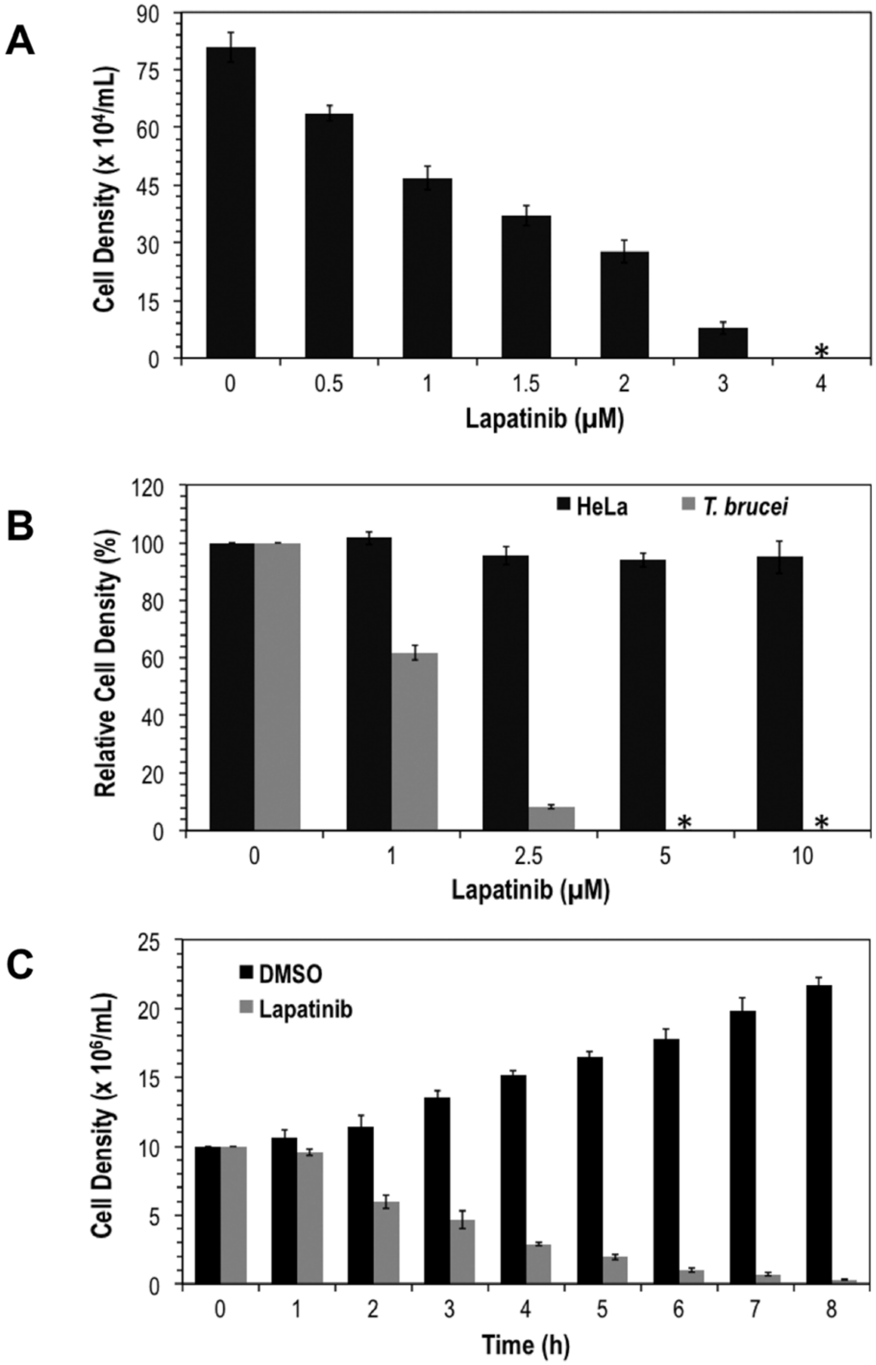

Figure 2. Lapatinib, AEE788 and canertinib kill bloodstream *T. brucei*. Bloodstream form *T. brucei* (initial cell density of 2×10^3 cells/ml) were cultured in 24-well plates for 48 h with either DMSO (control) or different concentrations of drug. Cells were counted with a hemocytometer and the graphs plotted. Data are mean $\pm$ standard deviation obtained from two independent experiments performed in duplicate. (***A***). Effect of lapatinib on growth of *T. brucei*. (***B***). Comparison of growth inhibitory effect of lapatinib on *T. brucei* to HeLa cells. HeLa and *T. brucei* cells were cultured for 48 h with variable concentrations of lapatinib. Relative cell density represents the live cells expressed as percentages of the control (*i.e.*, DMSO-treated) experiment. (***C***) Trypanosome kill assay. Time-course of trypanocidal effect of lapatinib. Trypanosomes (10^6 cells/ml) were treated with DMSO (solvent) or lapatinib (10 γM) for 8 h: viable cells were counted every hour, and plotted for each time point.
doi:10.1371/journal.pone.0056150.g002

differences between bound and unbound states, E_{int} is the ligand internal strain, ΔS_{Tor} is its conformational entropy loss upon binding, T = 300 K, and α_i are ligand- and protein-independent constants that have been previously optimized on a diverse screening benchmark.

Results

Lapatinib, Canertinib and AEE788 Inhibit Replication of *T. brucei* at Low Micromolar Concentrations

In the African trypanosome, endocytosis of transferrin, which is necessary for replication [41], is inhibited by Tyrphostin A47, a

pan-Tyr kinase inhibitor that also kills the parasite [24]. This data suggests that chemical scaffolds of drugs that inhibit protein Tyr kinases have potential to be ''re-purposed'' for anti-trypanosome lead drug discovery.

Lapatinib, canertinib and AEE788 (Fig. 1) are Tyr kinase inhibitors that have passed phase I clinical trials, at least, for use in humans [42–45]. In addition, the chemistry for synthesis of these drugs is well established and streamlined for optimization [11,46,47]. To evaluate effects of these drugs on *T. brucei,* axenically cultured bloodstream cells were exposed to different amounts of the compounds, and checked for viability. Lapatinib killed *T. brucei* (GI_{50} = 1.5 μMγ(Fig. 2A)). Similarly, canertinib and AEE788 killed the trypanosome with an GI_{50}'s of 2 μM, and 3 μM, respectively (data not presented). Ability of lapatinib to interfere with pro-survival pathways (*i.e.*, kill within the half-time of cell replication) in *T. brucei* was tested directly. When cells (10^6/ml) were incubated with the drug the survival half-life was 3 h (Fig. 2C), which is shorter than the cell division time of 6–8 h.

Toxicity of lapatinib against *T. brucei* was compared to its effect on a human HeLa cell line. At 5 μM or 10 μM all trypanosomes were killed but the human cells survived (Fig. 2B), presumably because growth of HeLa is not dependent on a hyperactive EGFR/HER2 [9,12]. Selectivity of lapatinib against the trypanosome is similar to that observed for the drug when tumor cells are compared to untransformed (non-sensitive) human cells [11,48].

Lapatinib, Canertinib and AEE788 Bind Five Trypanosome Protein Kinases

The respectable whole cell potency (*i.e.*, in a phenotypic screen) of lapatinib, canertinib and AEE788 against *T. brucei* (Fig. 2) could not be explained by inhibition of EGFR and/or VEGFR kinases, because the trypanosome genome does not encode classic receptor Tyr kinases [49]. Therefore, we postulated that the drugs had alternate protein targets in the trypanosome.

To identify the trypanosome targets of the drugs we used them as chemical tools. In our approach, a cell lysate from *T. brucei* was adsorbed to an ATP-sepharose column. After extensive washing of the ATP-affinity column with buffers containing high salt (*i.e.*, 1 M KCl) and/or NAD^+ (100 μM), the drugs (100 μM) were used to elute proteins remaining on the column. Proteins eluted were identified by mass spectrometry and bioinformatic analysis [29,50,51]. The profile of proteins eluted with drugs after the high salt wash is shown in Fig. 3B–D, and protein kinases identified are listed in Table 1. In preliminary studies, the profile of eluted proteins was very similar for the 1 or 10 or 100 μM drug concentrations. The higher concentration of drug eluted more proteins from the affinity column, judging from the intensity of the silver-stained bands and the number proteins identified by mass spectrometry (see Table S2 and Table S3); it was used for our studies.

Only five protein kinases, out of a possible 186 in the trypanosome genome [19,52] bound the three drugs. Tb927.3.1570, Tb927.4.1580 and Tb927.5.800 were eluted with AEE788, canertinib and lapatinib (Table 1). Tb10.60.3140 was eluted by both canertinib and lapatinib, whereas Tb10.61.1880 was only eluted by canertinib. Three lapatinib-binding protein kinases (TbLBPKs) have been studied earlier; TbLBPK1 (termed a Tousled-like kinase) [53,54], TbLBPK-2 (TbCK1.2 [55]) and TbLBPK4 (TbGSK3γ [56]). Non-kinase ATP-binding proteins detected in our dataset are available in Table S2. We focused primarily on protein kinases because lapatinib binds that class of enzymes in human cells [57].

Inclusion of NAD^+ in Washes of the Affinity Column Alters Profile of Protein Kinases Eluted by Drugs

Cells contain purine cofactors and nucleotides, including NAD^+, whose structure overlaps significantly that of ATP, which is a substrate of protein kinases. Recognizing the possibility that protein kinases may bind weakly to NAD^+, we explored a second strategy for our chemical proteomics study. Here, after washing the affinity column with the high salt buffer, we eluted the matrix with NAD+ (100 μM) before desorbing the residual proteins with drugs (see Fig. 3). Protein kinases in drug eluates are presented in Table 3.

After the NAD^+ wash, only two protein kinases were eluted from the affinity column. Tb927.4.5180 was eluted with lapatinib, and Tb927.5.800 was found in the AEE788 eluate. Canertinib failed to elute any protein kinase with this revised protocol. One explanation for these data, compared to proteins eluted without the NAD^+ wash (compare Table 1 and Table 3),

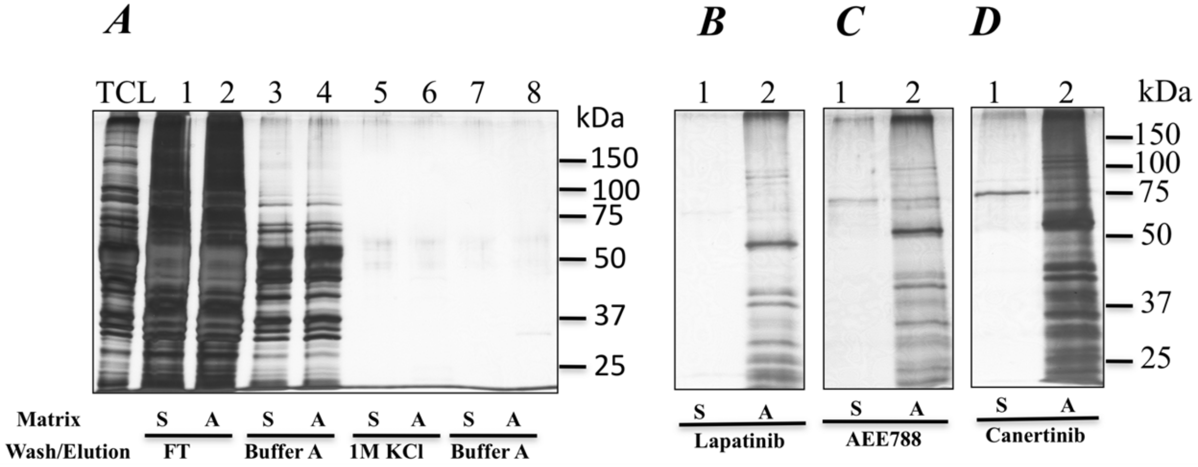

Figure 3. Drug elution of *T. brucei* protein kinases using an ATP-affinity resin. Proteins in total cell lysate from *T. brucei* were bound on ATP resin. Sepharose 4B resin was used as control. Unbound proteins (Flow through) were recovered and resin was washed sequentially with buffer A and buffer A containing 1 M KCl (Panel A). Bound proteins were eluted with lapatinib (100 γM), or AEE788 (100 γM) or canertinib (100 γM) (Panel B, C and D, respectively). Proteins were visualized by silver staining. Matrix label, ***A*** is ATP-sepharose, and ***S*** is Sepharose 4B.
doi:10.1371/journal.pone.0056150.g003

Table 3. Proteins eluted with lapatinib, or AEE788 or canertinib after 1 M KCl and NAD+ washes of the affinity chromatography column.

Protein	Description	Lapatinib	AEE788	Canertinib
		Total peptides	Total peptides	Total peptides
Tb927.4.5180	Protein Kinase putative	6	0	0
Tb927.5.800	Protein Kinase	0	3	0

Proteins listed are detected at least twice in three separate affinity chromatography/mass spectrometry studies.
doi:10.1371/journal.pone.0056150.t003

is that some protein kinases were eluted by NAD^+. We evaluated this hypothesis by analyzing the NAD^+ eluate for protein kinases, and found Tb927.5.800, Tb10.70.2070, Tb10.61.1880, and Tb10.61.3140. We conclude that NAD^+ can elute select trypanosome protein kinases from an ATP-affinity column, and alter the profile of proteins eluted by drugs from the ATP-affinity matrix.

Binding of Lapatinib or NAD^+ to TbLBPKs is Affirmed by Molecular Modeling

The affinity chromatography data indicates that lapatinib, as a model inhibitor, binds to 4 protein kinases (Table 1). Yet an NAD^+ wash of the column left Tb427.4.5180 (TbLBPK1) as the sole kinase eluted by the drug (Table 3). A similar situation is observed with AEE788 that binds three TbLBPKs initially but after the NAD^+ elution only desorbs TbLBPK2 (compare Table 1 and Table 3).

There are two possible explanations for these observations. One possibility is that NAD^+ binds to an ATP-binding "passenger protein" that associates with some TbLBPKs that do not directly bind the affinity column. As a result, removal of the passenger protein with NAD+ also elutes those TbLBPKs leaving only those that did not bind the passenger protein. An alternative explanation is that TbLBPKs bind both ATP and NAD^+ due to structural similarities between the two small molecules. In this view, NAD^+ elutes proteins from the affinity column leaving only those that bind tightest to ATP. At this point only drugs with significant affinity for a TbLBPK can elute the protein from the affinity matrix. Using computational approaches, we have evaluated, ahead of biochemical analysis, whether either any of these ideas have merit, by testing whether TbLBPKs bind lapatinib or NAD^+ or ATP.

Lapatinib binds and/or stabilizes a specific inactive conformation of the kinase domain in its human targets [58–60]; this conformation is characterized by (i) broken conserved Lys-Glu salt bridge, (ii) outward movement of the αC helix, and (iii) flipped conserved Phe at the base of the activation loop (DFG motif). Because of the inherent plasticity of the kinase domain, this conformation can also be spontaneously adopted or induced in many other kinases: according to our estimates, up to 45% of kinases in the PDB are found at least once in a lapatinib-compatible conformation. For the study of the structural determinants of lapatinib interaction with TbLBPKs 1–4, it is important that these target kinases are modeled in the lapatinib-compatible conformation. The closest homology templates for TbLBPK1 and 3 (but not TbLBPK2 or 4) have lapatinib-compatible X-ray structures (Fig. 4). As a trade-off between conformational relevance of the homology modeling template and the level of its sequence homology to the target(s), we chose to model TbLBPKs from human EGFR in several conformational variants [59].

The models constructed as described in Materials and Methods helped to elucidate possible structural basis of TbLBPK interaction with lapatinib and AEE788 and to identify amino residues that shape the drug-binding pocket (Fig. 5). This computational model confirmed that TbLBPKs can adopt the lapatinib-compatible backbone conformation, and that in all four cases, pocket residues are positioned favorably for lapatinib binding (Fig. 5A).

Docking scores for different kinase•drug complexes were consistent with the affinity chromatography data (Table 1 and Table 3): lapatinib, AEE788, and canertinib scored most favorably in the models of TbLBPK1, TbLBPK2, and TbCBPK1, respectively (Fig. 6A). The best poses for the drug binding to the protein kinases are depicted in Fig. 6B. In comparing the chromatography data with the models we found than an arbitrarily chosen ICM score cutoff of −42 separates drug/kinases complexes that are resistant to NAD^+ elution from the ATP-affinity column. For canertinib, the results obtained make for interesting discussion: TbCBPK1 docks best to canertinib (among the three drugs tested). However the ICM score was less than 42 for all three drugs, suggesting that TbCBPK1 will not be eluted by any drug from the affinity column after an NAD^+ wash (Fig. 6A). These data suggest that the models could have predictive value: a ligand•TbLBPK complex that receives a score −41 or less is not likely to be eluted from the ATP-affinity column after an NAD^+ wash (Table 3 and Table 4).

How could one explain the decreased docking scores, as compared to TbLBPK1, for TbLBPK2 and TbLBPK4? Here the decrease in predicted binding affinity of lapatinib may be explained by a missing residue in the hinge region (as compared to human EGFR), which leads to non-optimal orientation of the backbone amide groups. In the case of TbLBPK3, the likely reason for a docking score decrease is a modified environment for the fluorophenyl moiety of the drug, which, for example, includes a non-conservative substitution of Leu in EGFR for Tyr in TbLBPK3 (β_4 strand of the kinase) (Fig. 5).

We also tested whether TbLBPKs bind NAD^+ as postulated earlier. For this objective, we constructed models of TbLBPKs in the ATP-compatible conformation and docked ATP or NAD^+ into the models. Due to high structural similarity between ATP and NAD^+, both molecules fit well in the binding pockets and make similar hydrogen bonds with the hinge region and with the conserved Lys in the β3 strand of the kinase. However, entropic and solvation electrostatics penalties for the larger and more flexible NAD^+ molecule led to better binding scores for ATP (Fig. 7). These results indicate that high concentrations of NAD^+ are likely to compete with ATP for binding to TbLBPK and may wash some TbLBPKs off the ATP-affinity column. This prediction is confirmed by detection of Tb927.10.5140, Tb927.5.800 (TbLBPK2), and Tb10.61.1880 in the NAD^+ eluate from the ATP-affinity column (not presented). Interestingly, one protein

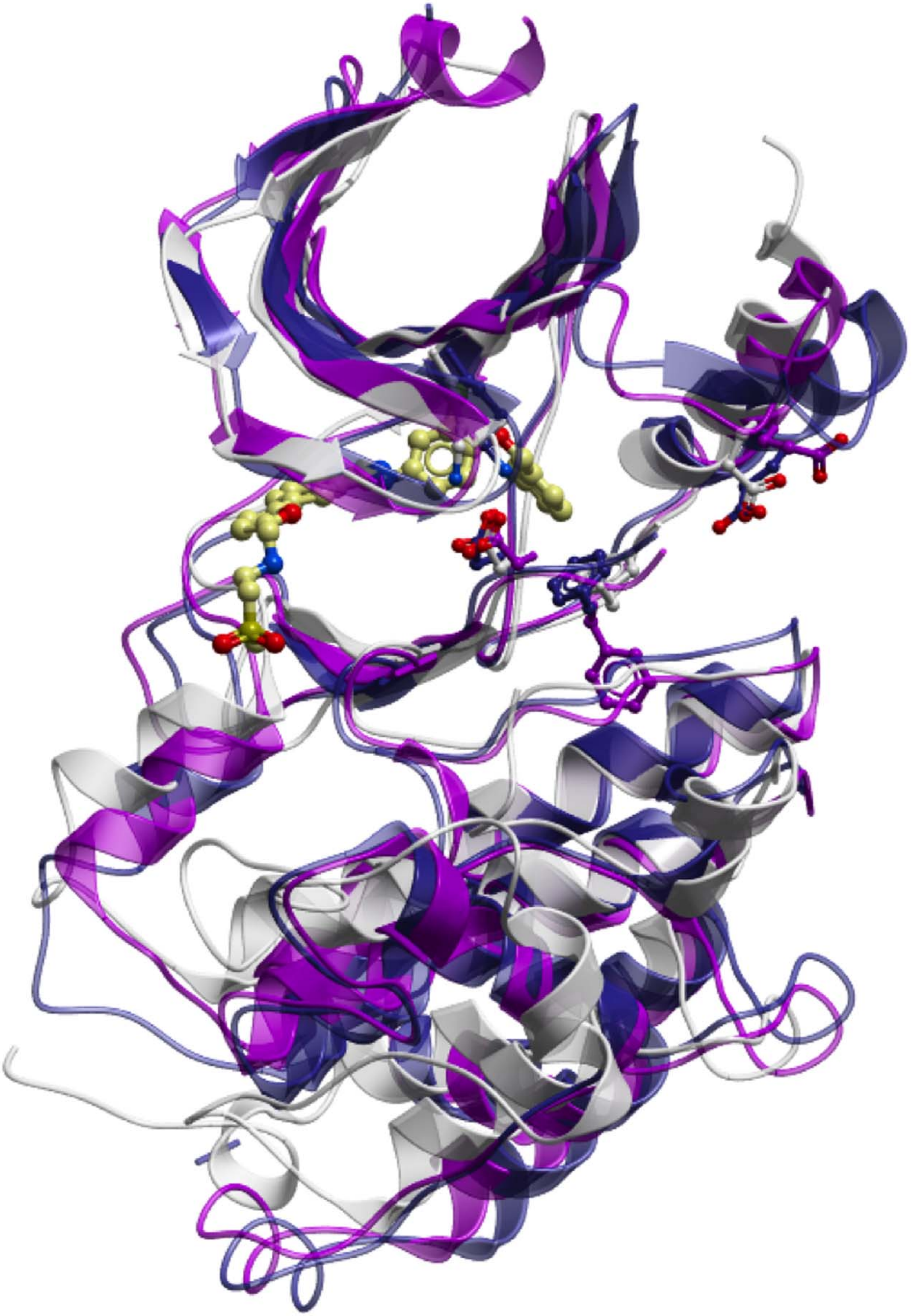

Figure 4. Closest homology templates for TbLBPK1 (magenta) and TbLBPK3 (navy) are crystallographically observed in a conformation similar to that of lapatinib-bound human EGFR (white).
doi:10.1371/journal.pone.0056150.g004

kinase eluted by NAD^+ *i.e.*, Tb927.10.5140 was not eluted by any of the drugs used in this study.

Discussion

New Chemical Entities for Anti-Trypanosome for Hit-to-Lead Studies

Current anti-trypanosome chemotherapies are difficult to deliver safely and some can be very toxic [1]. For example, in late stage human African trypanosomiasis (HAT) when the parasite has entered the central nervous system, the arsenical drug melarsoprol is the frontline treatment despite its toxicity (5% of patients die from side effects). Consequently, a pressing need exists for new therapeutics with oral bioavailability and good safety profile.

We discovered that endocytosis of transferrin by bloodstream *T. brucei* is inhibited by Tyrphostin A47 [24,25], a pan-inhibitor of protein Tyr kinases that also kills the parasite (Subramanya and Mensa-Wilmot, unpublished). We inferred from these data that inhibitors of *T. brucei* protein Tyr kinases or dual-specificity protein kinases (trypanosomes lack classic Tyr kinases (*e.g.*, EGFR)) might be valuable as "hits" for anti-trypanosome lead drug discovery. After performing a "focused screen" of investigational or approved Tyr kinase drugs, we settled on the 4-anilinoquinazolines carnetinib (CI-1033) [14,46,61] and lapatinib (GW572016) [42,58], and the pyrrolopyrimidine AEE788 [12,62] (Fig. 1) as possible "hits" for anti-trypanosome lead discovery. Canertinib, lapatinib, and AEE788 killed bloodstream *T. brucei*, with GI_{50} of 3 μM or less (Fig. 2).

The biological importance of three TbLBPKs has been characterized in RNA interference studies; TbLBPK1 (previously named Tousled-like kinase) [53,54], TbLBPK2 (TbCK1.2 [55]) and TbLBPK4 (TbGSK3γ [56]) are essential for viability of bloodstream *T. brucei.* Our discovery that these essential enzymes bind 4-anilinoquinazoline drugs that kill the parasite suggests that this class of small molecules is a valuable starting point for medicinal chemistry optimization [63–65] as anti-trypanosomal agents. That work is in progress.

A General Method for Discovering Targets of Kinase Inhibitor Drugs

Lapatinib kills bloodstream *T. brucei* with a GI_{50} in the low micromolar range (Fig. 2). To advance studies aimed at optimizing anti-trypanosome properties of lapatinib, it is important to identify the protein kinases that recognize the drug. This task is made challenging by the absence of EGFR/HER2 family of kinases in *T. brucei* [19,52]. Chemical proteomics [66] involving drug affinity chromatography seemed a reasonable path to discover the

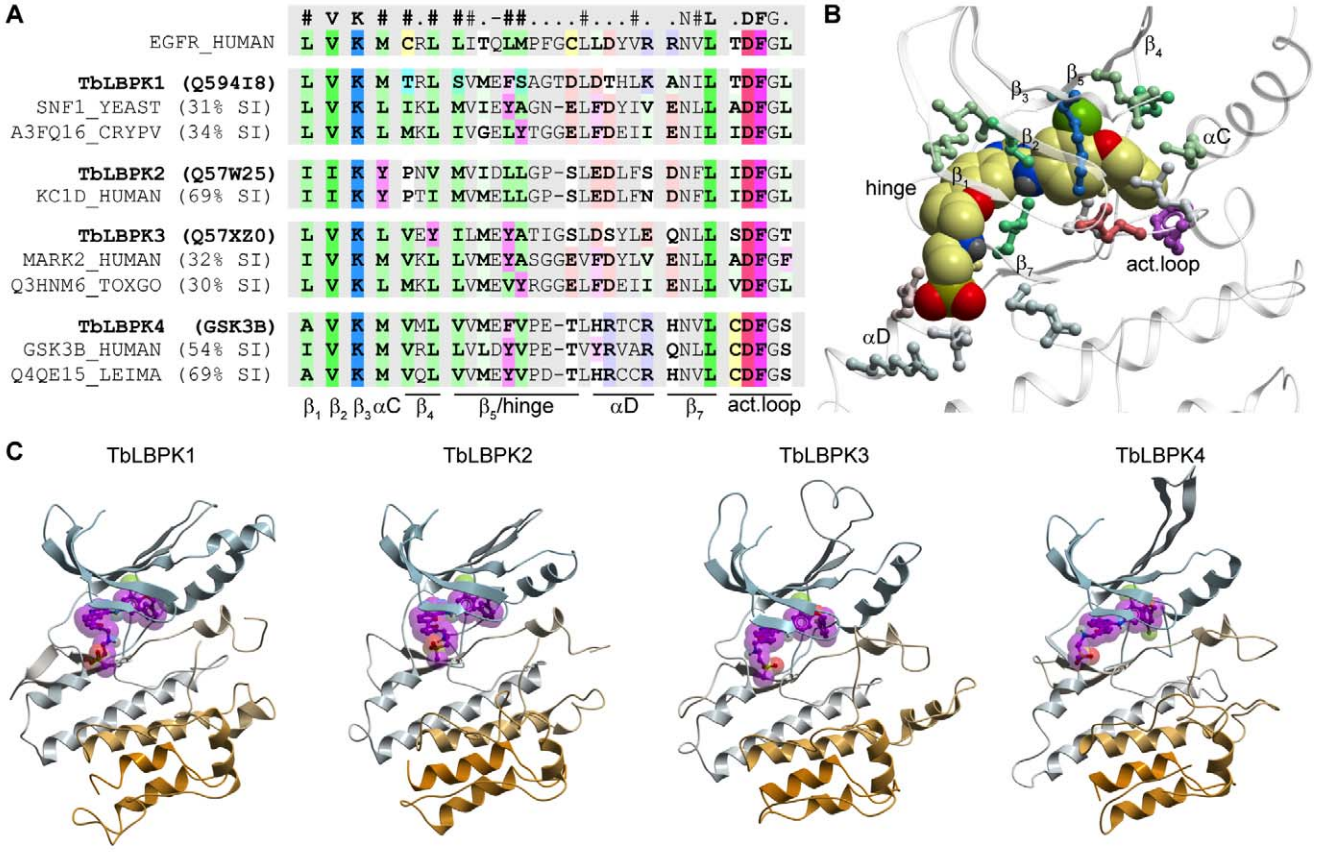

Figure 5. Homology modeling of *T. brucei* lapatinib-binding kinases using structures of human EGFR as templates. (*A*) Alignment of lapatinib binding site residues. (*B*) Binding site structures colored according to the alignment. (*C*) Models of TbLBPK's1-4 complexed with lapatinib.
doi:10.1371/journal.pone.0056150.g005

Table 4. An NAD+ wash of affinity columns alters ligand selection of target proteins.

Protein	Lapatinib		AEE788		Canertinib	
	KCL	KCl/NAD+	KCL	KCl/NAD+	KCL	KCl/NAD+
Tb927.3.1570	+	−	+	−	+	−
Tb927.4.5180	+	+	+	−	+	−
Tb927.5.800	+	−	+	+	+	−
Tb10.61.1880	−	−	−	−	+	−
Tb10.61.3140	+	−	−	−	+	−

doi:10.1371/journal.pone.0056150.t004

trypanosome targets for lapatinib. Nevertheless, we opted to develop a more versatile protocol that would not require the use of specialized matrices containing covalently attached kinase inhibitors (*e.g.*, kinobeads) [66,67].

Since all protein kinases can bind to ATP, one could use the trinucleotide as a universal ligand for that class of enzymes (as well as other ATP-binding proteins). Therefore we used an ATP-affinity column, instead of covalently attaching the drugs directly to a matrix. After that we bound a lysate of total cellular proteins to the affinity column, we washed it stringently to disrupt non-specific protein-matrix interactions, and then eluted with drugs (*e.g.*, lapatinib or canertinib or AEE788). Proteins eluted by the drugs were identified with mass spectrometry and bioinformatic approaches (see *Materials and Methods*) (see Table 1). Our approach can be used to discover cellular targets of drugs that interfere with ATP•protein interactions/metabolism. Indeed we have used the protocol to discover trypanosome protein targets of drugs other than those reported here (R. Behera and K. Mensa-Wilmot, in preparation.). A related technique was published while this manuscript was being written [68].

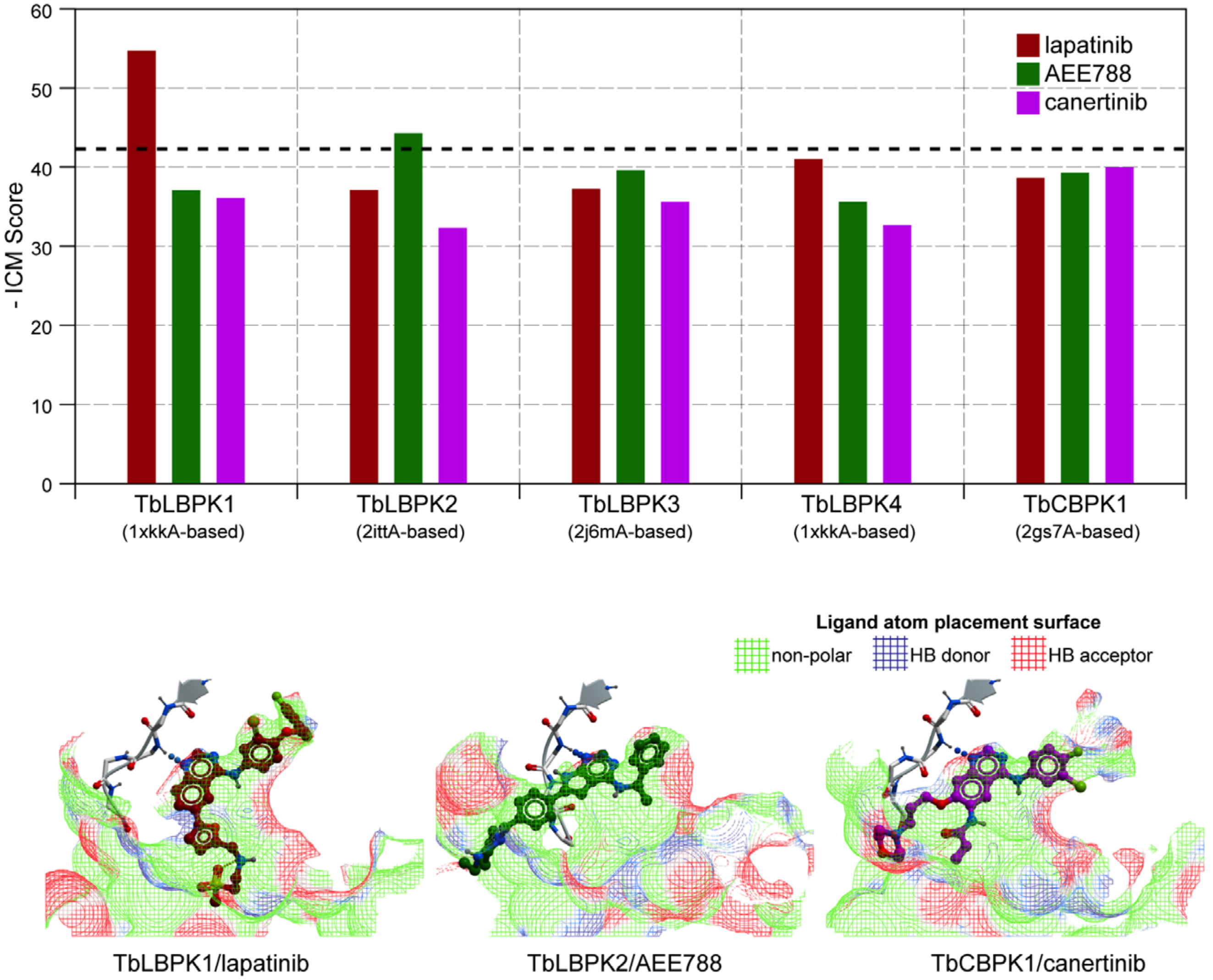

Figure 6. Best models of TbLBPK•drug complexes are consistent with affinity chromatography elution data. (*A*) Predicted ICM binding scores of lapatinib (red), AEE788 (green) and canertinib (magenta) in the binding pockets of the protein kinases. The dotted line represents a hypothetical cutoff for kinase elution by drug after an NAD+ wash of the affinity column. (*B*) Predicted binding poses for lapatinib (red), AEE788 (green) and canertinib (magenta) to their highest affinity protein kinases; TbLBPK1, TbLBPK2, and TbCBPK1, respectively. Kinase hinge region is shown in ribbon, ligand atom placement surface is represented by a wire mesh and colored according to its binding properties.
doi:10.1371/journal.pone.0056150.g006

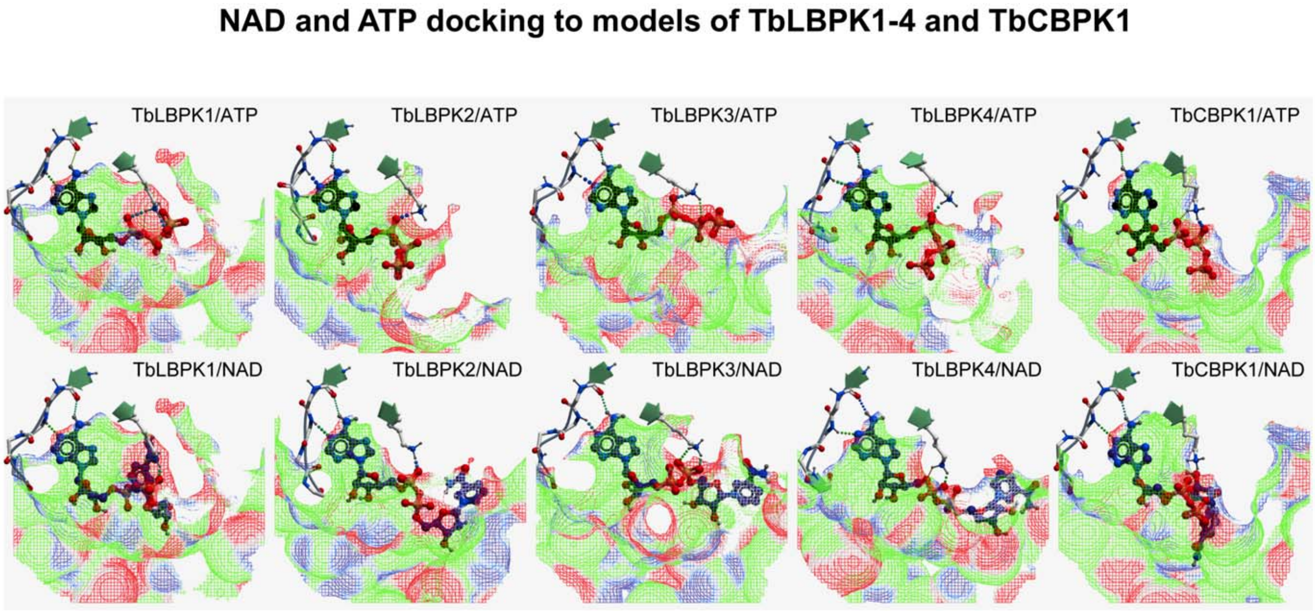

Figure 7. Docking of NAD^+ and ATP to homology models of TbLBPKs and TbCBPK1.
doi:10.1371/journal.pone.0056150.g007

It is worth mentioning that the protocol devised for this study parallels the situation that a drug encounters intracellularly where ATP is present at millimolar levels. Our protocol replicates this scenario by adding a cell lysate to an ATP affinity column first, before introducing a drug to elute the target protein. ATP concentration on the affinity column is estimated to be about 1–10 mM [69]. To elute specific proteins, one can use as low as 1 μM of drug and titrate the concentration up as desired until one detects clear bands in silver-stained polyacrylamide gels (see Fig. 2 for example). The latter condition is important for successful identification of proteins by mass spectrometry.

Four Trypanosome Proteins are Likely to Adopt a Lapatinib-Compatible Backbone Conformation

Lapatinib is one of the most selective among 38 kinase inhibitors tested against 317 human enzymes [57]. In addition to the Tyr kinases EGFR/HER2, the drug binds with appreciable affinity to three other protein kinases, namely STK10/LOK, RIPK2, and STK2/SLK [70–72]. This high selectivity of lapatinib is partially due to a specific conformation of the kinase domain bound by the drug [32]; it is characterized by (i) a broken conserved Lys-Glu salt bridge, (ii) a displaced αC helix, and (iii) a flipped conserved Phe at the base of the "activation loop" (*i.e.*, DFG motif) [64,73,74].

Up to 45% of kinases in the PDB, according to our estimates, can adopt a lapatinib-compatible backbone conformation (unpublished). Many structures of proteins with high sequence homology to TbLBPKs (Table 2) have features of the lapatinib-bound state, *e.g. Cryptosporidium parvum* calcium-dependent protein kinase 1 (with sequence similarity to TbLBPK1) and *Toxoplasma gondii* calcium-dependent protein kinase 3 (which has protein sequence similarity to TbLBPK3). Modeling of TbLBPKs from a lapatinib-compatible EGFR template (PDB 1xkk) produced structures that are free of steric conflicts or energetically strained regions (Fig. 4). These modeling data support conclusions that trypanosome lapatinib-binding proteins can adopt a lapatinib-compatible backbone as one of the equilibrium conformations for their kinase domains (Fig. 5).

Supporting Information

Table S1 Properties of Peptides Used to Identify Protein Protein Kinases.
(DOCX)

Table S2 Proteins eluted with lapatinib or AEE788 or canertinib (100 γM each) after 1 M KCl wash of ATP-affinity column. Only proteins for which two or more peptides were identified are listed. Proteins eluted with DMSO (1%) (solvent for the drugs) were identified in a separate analysis and eliminated from the drug-eluted proteins in the table.
(XLS)

Table S3 Proteins eluted with 10 γM lapatinib after 1 M KCl wash of ATP-affinity column. Proteins containing 2 or more peptides are listed.
(XLSX)

Acknowledgments

We thank Timothy Haystead (Duke University) for invaluable discussions, and gifts of ATP-sepharose. For insightful comments on the manuscript, we than Paul Guyett (University of Georgia). We thank Lingling Shen for helpful discussion. We are grateful to Pfizer, GlaxoSmithKline and Novartis for gifts of canertinib, lapatinib, and AEE788. Work in KM-W lab was supported by grant AI076647 from the National Institutes of Health.

Author Contributions

Conceived and designed the experiments: SK RB IK YO MP RA KM-W. Performed the experiments: SK RB ST IK YO. Analyzed the data: SK RB ST IK YO MP RA KM-W. Wrote the paper: SK RB IK YO MP RA KM-W.

References

1. Barrett MP, Boykin DW, Brun R, Tidwell RR (2007) Human African trypanosomiasis: pharmacological re-engagement with a neglected disease. Br J Pharmacol 152: 1155–1171.
2. Brun R, Balmer O (2006) New developments in human African trypanosomiasis. Curr Opin Infect Dis 19: 415–420.
3. Sessa G, Raz V, Savaldi S, Fluhr R (1996) PK12, a plant dual-specificity protein kinase of the LAMMER family, is regulated by the hormone ethylene. Plant Cell 8: 2223–2234.
4. Kentrup H, Becker W, Heukelbach J, Wilmes A, Schurmann A, et al. (1996) Dyrk, a dual specificity protein kinase with unique structural features whose activity is dependent on tyrosine residues between subdomains VII and VIII. J Biol Chem 271: 3488–3495.
5. Letwin K, Mizzen L, Motro B, Ben-David Y, Bernstein A, et al. (1992) A mammalian dual specificity protein kinase, Nek1, is related to the NIMA cell cycle regulator and highly expressed in meiotic germ cells. EMBO J 11: 3521–3531.
6. Parker LL, Atherton-Fessler S, Piwnica-Worms H (1992) p107wee1 is a dual-specificity kinase that phosphorylates p34cdc2 on tyrosine 15. Proc Natl Acad Sci U S A 89: 2917–2921.
7. Lemmon MA, Schlessinger J (2010) Cell signaling by receptor tyrosine kinases. Cell 141: 1117–1134.
8. Chen R, Gandhi V, Plunkett W (2006) A sequential blockade strategy for the design of combination therapies to overcome oncogene addiction in chronic myelogenous leukemia. Cancer Res 66: 10959–10966.
9. Sharma SV, Gajowniczek P, Way IP, Lee DY, Jiang J, et al. (2006) A common signaling cascade may underlie "addiction" to the Src, BCR-ABL, and EGF receptor oncogenes. Cancer Cell 10: 425–435.
10. Weinstein IB, Joe AK (2006) Mechanisms of disease: Oncogene addiction–a rationale for molecular targeting in cancer therapy. Nat Clin Pract Oncol 3: 448–457.
11. Lackey KE (2006) Lessons from the drug discovery of lapatinib, a dual ErbB1/2 tyrosine kinase inhibitor. Curr Top Med Chem 6: 435–460.
12. Traxler P, Allegrini PR, Brandt R, Brueggen J, Cozens R, et al. (2004) AEE788: a dual family epidermal growth factor receptor/ErbB2 and vascular endothelial growth factor receptor tyrosine kinase inhibitor with antitumor and antiangiogenic activity. Cancer Res 64: 4931–4941.
13. Barbarroja N, Torres LA, Rodriguez-Ariza A, Valverde-Estepa A, Lopez-Sanchez LM, et al. (2010) AEE788 is a vascular endothelial growth factor receptor tyrosine kinase inhibitor with antiproliferative and proapoptotic effects in acute myeloid leukemia. Exp Hematol.
14. Slichenmyer WJ, Elliott WL, Fry DW (2001) CI-1033, a pan-erbB tyrosine kinase inhibitor. Semin Oncol 28: 80–85.
15. Rixe O, Franco SX, Yardley DA, Johnston SR, Martin M, et al. (2009) A randomized, phase II, dose-finding study of the pan-ErbB receptor tyrosine-kinase inhibitor CI-1033 in patients with pretreated metastatic breast cancer. Cancer Chemother Pharmacol 64: 1139–1148.
16. Trinks C, Djerf EA, Hallbeck AL, Jonsson JI, Walz TM (2010) The pan-ErbB receptor tyrosine kinase inhibitor canertinib induces ErbB-independent apoptosis in human leukemia (HL-60 and U-937) cells. Biochem Biophys Res Commun 393: 6–10.
17. Nett IR, Martin DM, Miranda-Saavedra D, Lamont D, Barber JD, et al. (2009) The phosphoproteome of bloodstream form trypanonosoma brucei, causative agent of African sleeping sickness. Mol Cell Proteomics.
18. Nett IR, Davidson L, Lamont D, Ferguson MA (2009) Identification and specific localization of tyrosine-phosphorylated proteins in Trypanosoma brucei. Eukaryot Cell 8: 617–626.
19. Parsons M, Worthey EA, Ward PN, Mottram JC (2005) Comparative analysis of the kinomes of three pathogenic trypanosomatids: Leishmania major, Trypanosoma brucei and Trypanosoma cruzi. BMC Genomics 6: 127.
20. Lochhead PA, Sibbet G, Kinstrie R, Cleghon T, Rylatt M, et al. (2003) dDYRK2: a novel dual-specificity tyrosine-phosphorylation-regulated kinase in Drosophila. Biochem J 374: 381–391.
21. Lim WA, Pawson T (2010) Phosphotyrosine signaling: evolving a new cellular communication system. Cell 142: 661–667.
22. Wheeler-Alm E, Shapiro SZ (1992) Evidence of Tyrosine Kinase Activity in the Protozoan Parasite Trypanosoma brucei JProtozool 39: 413–416.
23. Hamadien M, Bakhiet M, Harris RA (2000) Interferon-gamma induces secretion of trypanosome lymphocyte triggering factor via tyrosine protein kinases. Parasitology 120 (Pt 3): 281–287.
24. Subramanya S, Mensa-Wilmot K (2010) Diacylglycerol-stimulated endocytosis of transferrin in trypanosomatids is dependent on tyrosine kinase activity. PLoS One 5: e8538.
25. Subramanya S, Hardin CF, Steverding D, Mensa-Wilmot K (2009) Glycosylphosphatidylinositol-specific phospholipase C regulates transferrin endocytosis in the African trypanosome. Biochem J 417: 685–694.
26. Hirumi H, Hirumi K (1994) Axenic culture of African trypanosome bloodstream forms. ParasitolToday 10: 80–84.
27. Olaussen KA, Commo F, Tailler M, Lacroix L, Vitale I, et al. (2009) Synergistic proapoptotic effects of the two tyrosine kinase inhibitors pazopanib and lapatinib on multiple carcinoma cell lines. Oncogene 28: 4249–4260.
28. Deutsch EW, Mendoza L, Shteynberg D, Farrah T, Lam H, et al. (2010) A guided tour of the Trans-Proteomic Pipeline. Proteomics 10: 1150–1159.
29. Rauch A, Bellew M, Eng J, Fitzgibbon M, Holzman T, et al. (2006) Computational Proteomics Analysis System (CPAS): an extensible, open-source analytic system for evaluating and publishing proteomic data and high throughput biological experiments. J Proteome Res 5: 112–121.
30. Higdon R, Hogan JM, Kolker N, van Belle G, Kolker E (2007) Experiment-specific estimation of peptide identification probabilities using a randomized database. OMICS 11: 351–365.
31. Myers T, Law W, Eng JK, McIntosh M (2007) Installation and use of the Computational Proteomics Analysis System (CPAS). Curr Protoc Bioinformatics Chapter 13: Unit 13 15.
32. Wood ER, Truesdale AT, McDonald OB, Yuan D, Hassell A, et al. (2004) A Unique Structure for Epidermal Growth Factor Receptor Bound to GW572016 (Lapatinib). Cancer Res 64: 6652–6659.
33. Kufareva I, Rueda M, Katritch V, participants GD, Stevens RC, et al. (2011) Status of GPCR modeling and docking as reflected by community wide GPCR Dock 2010 assessment. Structure accepted.
34. Abagyan R, Batalov S, Cardozo T, Totrov M, Webber J, et al. (1997) Homology modeling with internal coordinate mechanics: deformation zone mapping and improvements of models via conformational search. Proteins Suppl 1: 29–37.
35. Rost B (1999) Twilight zone of protein sequence alignments. Protein Eng 12: 85–94.
36. Abagyan RA, Totrov MM, Kuznetsov DA (1994) Icm: A New Method For Protein Modeling and Design: Applications To Docking and Structure Prediction From The Distorted Native Conformation. J Comp Chem 15: 488–506.
37. Abagyan R, Totrov M (1994) Biased probability Monte Carlo conformational searches and electrostatic calculations for peptides and proteins. Journal of molecular biology 235: 983–1002.
38. Arnautova YA, Abagyan RA, Totrov M (2010) Development of a new physics-based internal coordinate mechanics force field and its application to protein loop modeling. Proteins: Structure, Function, and Bioinformatics 79: 477–498.
39. Laskowski RA, MacArthur MW, Moss DS, Thornton JM (1993) PROCHECK: a program to check the stereochemical quality of protein structures,. J Appl Cryst 26: 283–291.
40. Schapira M, Totrov M, Abagyan R (1999) Prediction of the binding energy for small molecules, peptides and proteins. Journal of Molecular Recognition 12: 177–190.
41. Schell D, Borowy NK, Overath P (1991) Transferrin is a growth factor for the bloodstream form of Trypanosoma brucei. Parasitol Res 77: 558–560.
42. Burris HA, 3rd, Hurwitz HI, Dees EC, Dowlati A, Blackwell KL, et al. (2005) Phase I safety, pharmacokinetics, and clinical activity study of lapatinib (GW572016), a reversible dual inhibitor of epidermal growth factor receptor tyrosine kinases, in heavily pretreated patients with metastatic carcinomas. J Clin Oncol 23: 5305–5313.
43. Lu C, Kamat AA, Lin YG, Merritt WM, Landen CN, et al. (2007) Dual targeting of endothelial cells and pericytes in antivascular therapy for ovarian carcinoma. Clin Cancer Res 13: 4209–4217.
44. Heymach JV, Nilsson M, Blumenschein G, Papadimitrakopoulou V, Herbst R (2006) Epidermal growth factor receptor inhibitors in development for the treatment of non-small cell lung cancer. Clin Cancer Res 12: 4441s–4445s.
45. Arora A, Scholar EM (2005) Role of tyrosine kinase inhibitors in cancer therapy. J Pharmacol Exp Ther 315: 971–979.
46. Smaill JB, Showalter HD, Zhou H, Bridges AJ, McNamara DJ, et al. (2001) Tyrosine kinase inhibitors. 18. 6-Substituted 4-anilinoquinazolines and 4-anilinopyrido[3,4-d]pyrimidines as soluble, irreversible inhibitors of the epidermal growth factor receptor. J Med Chem 44: 429–440.
47. Xia W, Mullin RJ, Keith BR, Liu LH, Ma H, et al. (2002) Anti-tumor activity of GW572016: a dual tyrosine kinase inhibitor blocks EGF activation of EGFR/erbB2 and downstream Erk1/2 and AKT pathways. Oncogene 21: 6255–6263.
48. Kim JW, Kim HP, Im SA, Kang S, Hur HS, et al. (2008) The growth inhibitory effect of lapatinib, a dual inhibitor of EGFR and HER2 tyrosine kinase, in gastric cancer cell lines. Cancer Lett 272: 296–306.
49. Berriman M, Ghedin E, Hertz-Fowler C, Blandin G, Renauld H, et al. (2005) The Genome of the African Trypanosome Trypanosoma brucei. Science 309: 416–422.
50. Panigrahi AK, Ogata Y, Zikova A, Anupama A, Dalley RA, et al. (2009) A comprehensive analysis of Trypanosoma brucei mitochondrial proteome. Proteomics 9: 434–450.
51. Gafken PR (2009) An overview of the qualitative analysis of phosphoproteins by mass spectrometry. Methods in molecular biology 527: 159–172, ix.
52. El-Sayed NM, Myler PJ, Bartholomeu DC, Nilsson D, Aggarwal G, et al. (2005) The Genome Sequence of Trypanosoma cruzi, Etiologic Agent of Chagas Disease. Science 309: 409–415.
53. Li Z, Umeyama T, Wang CC (2008) The chromosomal passenger complex and a mitotic kinesin interact with the Tousled-like kinase in trypanosomes to regulate mitosis and cytokinesis. PLoS One 3: e3814.
54. Li Z, Lee JH, Chu F, Burlingame AL, Gunzl A, et al. (2008) Identification of a novel chromosomal passenger complex and its unique localization during cytokinesis in Trypanosoma brucei. PLoS One 3: e2354.

55. Urbaniak MD (2009) Casein kinase 1 isoform 2 is essential for bloodstream form Trypanosoma brucei. Mol Biochem Parasitol 166: 183–185.
56. Ojo KK, Gillespie JR, Riechers AJ, Napuli AJ, Verlinde CL, et al. (2008) Glycogen synthase kinase 3 is a potential drug target for African trypanosomiasis therapy. Antimicrob Agents Chemother 52: 3710–3717.
57. Karaman MW, Herrgard S, Treiber DK, Gallant P, Atteridge CE, et al. (2008) A quantitative analysis of kinase inhibitor selectivity. Nat Biotech 26: 127–132.
58. Wood ER, Truesdale AT, McDonald OB, Yuan D, Hassell A, et al. (2004) A unique structure for epidermal growth factor receptor bound to GW572016 (Lapatinib): relationships among protein conformation, inhibitor off-rate, and receptor activity in tumor cells. Cancer Res 64: 6652–6659.
59. Yun CH, Boggon TJ, Li Y, Woo MS, Greulich H, et al. (2007) Structures of Lung Cancer-Derived EGFR Mutants and Inhibitor Complexes: Mechanism of Activation and Insights into Differential Inhibitor Sensitivity. Cancer Cell 11: 217–227.
60. Johnson LN (2009) Protein kinase inhibitors: contributions from structure to clinical compounds. Q Rev Biophys 42: 1–40.
61. Calvo E, Tolcher AW, Hammond LA, Patnaik A, de Bono JS, et al. (2004) Administration of CI-1033, an irreversible pan-erbB tyrosine kinase inhibitor, is feasible on a 7-day on, 7-day off schedule: a phase I pharmacokinetic and food effect study. Clin Cancer Res 10: 7112–7120.
62. Park YW, Younes MN, Jasser SA, Yigitbasi OG, Zhou G, et al. (2005) AEE788, a dual tyrosine kinase receptor inhibitor, induces endothelial cell apoptosis in human cutaneous squamous cell carcinoma xenografts in nude mice. Clin Cancer Res 11: 1963–1973.
63. Wissner A, Overbeek E, Reich MF, Floyd MB, Johnson BD, et al. (2003) Synthesis and structure-activity relationships of 6,7-disubstituted 4-anilinoquinoline-3-carbonitriles. The design of an orally active, irreversible inhibitor of the tyrosine kinase activity of the epidermal growth factor receptor (EGFR) and the human epidermal growth factor receptor-2 (HER-2). J Med Chem 46: 49–63.
64. Vema A, Panigrahi SK, Rambabu G, Gopalakrishnan B, Sarma JA, et al. (2003) Design of EGFR kinase inhibitors: a ligand-based approach and its confirmation with structure-based studies. Bioorg Med Chem 11: 4643–4653.
65. Hubbard RD, Dickerson SH, Emerson HK, Griffin RJ, Reno MJ, et al. (2008) Dual EGFR/ErbB-2 inhibitors from novel pyrrolidinyl-acetylenic thieno[3,2-d]pyrimidines. Bioorg Med Chem Lett 18: 5738–5740.
66. Brehmer D, Greff Z, Godl K, Blencke S, Kurtenbach A, et al. (2005) Cellular targets of gefitinib. Cancer Res 65: 379–382.
67. Bantscheff M, Eberhard D, Abraham Y, Bastuck S, Boesche M, et al. (2007) Quantitative chemical proteomics reveals mechanisms of action of clinical ABL kinase inhibitors. Nat Biotechnol 25: 1035–1044.
68. Duncan JS, Haystead TA, Litchfield DW (2012) Chemoproteomic characterization of protein kinase inhibitors using immobilized ATP. Methods Mol Biol 795: 119–134.
69. Haystead TA (2006) The purinome, a complex mix of drug and toxicity targets. Curr Top Med Chem 6: 1117–1127.
70. Walter SA, Cutler RE, Jr., Martinez R, Gishizky M, Hill RJ (2003) Stk10, a new member of the polo-like kinase kinase family highly expressed in hematopoietic tissue. J Biol Chem 278: 18221–18228.
71. Kaouass M, Audette M, Ramotar D, Verma S, De Montigny D, et al. (1997) The STK2 gene, which encodes a putative Ser/Thr protein kinase, is required for high-affinity spermidine transport in Saccharomyces cerevisiae. Mol Cell Biol 17: 2994–3004.
72. Goldammer T, Brunner RM, Kata SR, Womack JE (2006) Assignment of the bovine receptor-interacting serine-threonine kinase 2 gene (RIPK2) to BTA14 with somatic and radiation cell hybrids. Cytogenet Genome Res 112: 341C.
73. Roskoski R Jr (2004) Src protein-tyrosine kinase structure and regulation. Biochem Biophys Res Commun 324: 1155–1164.
74. Atwell S, Adams JM, Badger J, Buchanan MD, Feil IK, et al. (2004) A novel mode of Gleevec binding is revealed by the structure of spleen tyrosine kinase. The Journal of biological chemistry 279: 55827–55832.
